# N-3 Polyunsaturated Fatty Acids Protect against Alcoholic Liver Steatosis by Activating FFA4 in Kupffer Cells

**DOI:** 10.3390/ijms25105476

**Published:** 2024-05-17

**Authors:** Saeromi Kang, Jung-Min Koh, Dong-Soon Im

**Affiliations:** 1 Research Institute for Drug Development, Pusan National University, Busan 46241, Republic of Korea; saeromi85@gmail.com; 2 Division of Endocrinology and Metabolism, Asan Medical Center, College of Medicine, University of Ulsan, Seoul 05505, Republic of Korea; jmkoh@amc.seoul.kr; 3 Department of Basic Pharmaceutical Sciences, Graduate School, Kyung Hee University, Seoul 02447, Republic of Korea

**Keywords:** omega-3 fatty acid, hepatic steatosis, Kupffer cell, FFA4, GPR120, CpdA

## Abstract

Supplementation with fish oil rich in omega-3 polyunsaturated fatty acids (n-3 PUFAs) effectively reduces acute and chronic alcohol-induced hepatic steatosis. We aimed to find molecular mechanisms underlying the effects of n-3 PUFAs in alcohol-induced hepatic steatosis. Because free fatty acid receptor 4 (FFA4, also known as GPR120) has been found as a receptor for n-3 PUFAs in an ethanol-induced liver steatosis model, we investigated whether n-3 PUFAs protect against liver steatosis via FFA4 using AH7614, an FFA4 antagonist, and *Ffa4* knockout (KO) mice. N-3 PUFAs and compound A (CpdA), a selective FFA4 agonist, reduced the ethanol-induced increase in lipid accumulation in hepatocytes, triglyceride content, and serum ALT levels, which were not observed in *Ffa4* KO mice. N-3 PUFAs and CpdA also reduced the ethanol-induced increase in lipogenic sterol regulatory element-binding protein-1c expression in an FFA4-dependent manner. In Kupffer cells, treatment with n-3 PUFA and CpdA reversed the ethanol-induced increase in tumor necrosis factor-α, cyclooxygenase-2, and NLR family pyrin domain-containing 3 expression levels in an FFA4-dependent manner. In summary, n-3 PUFAs protect against ethanol-induced hepatic steatosis via the anti-inflammatory actions of FFA4 on Kupffer cells. Our findings suggest FFA4 as a therapeutic target for alcoholic hepatic steatosis.

## 1. Introduction

Alcohol consumption is causally related to more than 60 health conditions [1]. In 2016, 3.0 million deaths worldwide and 132 million disability-adjusted life years were attributed to alcohol consumption [2]. Alcoholic liver disease is a major cause of illness and death in affluent regions, such as Eastern Europe and sub-Saharan Africa, which have high per capita alcohol consumption [3]. Alcoholic fatty liver disease, characterized by excess accumulation of triglycerides in the liver, is an initial stage of liver disease and a reversible pathological condition [4]. However, prolonged and excessive alcohol consumption leads to advanced stages of alcoholic liver disease, including alcoholic steatohepatitis, fibrosis, cirrhosis, and hepatocarcinoma [5].

Dietary fish oil may be useful in preventing ethanol-induced fatty liver disease because prolonged omega-3 polyunsaturated fatty acids (n-3 PUFAs) supplementation ameliorated hepatic steatosis in patients with non-alcoholic fatty liver disease in many human trials [6,7]. In obese patients, upregulation of the sterol regulatory element-binding protein (SREBP)-1c, a key transcription factor for lipogenesis, and down-regulation of the peroxisome proliferator-activated receptor (PPAR)-α, a key transcription factor for fatty acid oxidation, occur in association with n-3 PUFA depletion and insulin resistance [8,9]. Alcoholic liver disease is associated with an insufficient intake of PUFAs. For example, a 24% decrease in total PUFAs has been reported in patients with alcohol-related liver damage [10]. In Rhesus monkeys, low dietary levels of PUFAs increase the risk of alcohol-induced liver disease [11]. Low levels of n-3 PUFAs, especially docosahexaenoic acid (DHA), are common in patients with alcoholic liver disease [12,13].

N-3 PUFAs effectively reduce liver fat in acute and chronic alcohol-induced steatosis models [14,15]. Furthermore, endogenously high levels of n-3 PUFAs alleviate ethanol-induced liver steatosis in fat-1 transgenic mice [16,17]. However, the precise molecular targets and action mechanisms of n-3 PUFAs remain ambiguous.

Pathogenic mechanisms of alcoholic liver disease involve complex interactions between the direct toxic effects of alcohol and its metabolites and the indirect upregulation of inflammatory responses. Activation of innate immune cells and inflammatory responses play key roles in the multifactorial pathophysiology of alcoholic liver disease [18]. The innate immune response elicited by Kupffer cells, hepatic macrophages, plays a vital role in early alcohol-induced liver injury by recognizing endotoxins in portal circulation and polarizing Kupffer cells toward a pro-inflammatory M1 phenotype [19,20].

Kupffer cells act as key players in alcoholic hepatic steatosis, and their depletion with gadolinium prevents alcoholic hepatic steatosis and liver injury [21,22]. In addition, the reduction in bacterial endotoxins via sterilization of the gut with antibiotics blocks early inflammation caused by alcohol. Tumor necrosis factor (TNF)-α also plays an important role in the development of early alcohol-induced liver steatosis and injury via the TNF-R1 pathway [23]. Therefore, chronic alcohol consumption increases the permeability of the gut, resulting in increased exposure to endotoxins, such as lipopolysaccharides (LPSs), which are membrane components of Gram-negative bacteria, via the portal vein. LPS-induced activation of Kupffer cells leads to the production of reactive oxygen species and TNF-α via Myd88-independent and TRIF/IRF3-dependent pathways, resulting in liver inflammation and injury [24,25,26,27,28]. TNF-α, mainly produced by sensitized Kupffer cells, is the most important cytokine in alcohol-induced liver injury [29]. In addition to its direct toxic effects on hepatocytes, TNF-α increases the de novo synthesis of fatty acids by upregulating SREBP-1c activity via the activation of a signal transducer and activator of transcription-3 [30,31]. Activation of macrophages leads to an increase in the levels of pro-inflammatory cytokines, such as TNF-α, interleukin (IL)-6, IL-8, IL-12, and IL-1β [19,32,33].

Recently, a G protein-coupled receptor, free fatty acid receptor 4 (FFA4, also known as GPR120), has been recognized as a receptor for n-3 PUFAs [34,35]. Its expression and anti-inflammatory functions in macrophages have been previously reported [34,35]. In particular, the anti-inflammatory effect of n-3 PUFA has been explained by FFA4/α-arrestin-mediated blockage of TGF-α activated kinase 1 activation in macrophages [34,35]. In Kupffer cells, FFA4 expression and its protective effects against ischemia-reperfusion injury have recently been reported [33].

Based on the above-mentioned studies, we hypothesized that the protective effects of n-3 PUFAs against alcoholic liver steatosis may involve an FFA4-mediated anti-inflammatory response in Kupffer cells. To assess the beneficial effects of n-3 PUFAs in relation to FFA4, we investigated the role of FFA4 in n-3 PUFA protection against alcoholic liver steatosis in combination with a selective FFA4 agonist, compound A (CpdA), and a selective antagonist, AH7614, using an ethanol-induced steatosis model and *Ffa4*-deficient mice.

## 2. Results

### 2.1. N-3 PUFAs Ameliorate Ethanol-Induced Steatosis and Liver Injury in Mice

**Figure 1:**
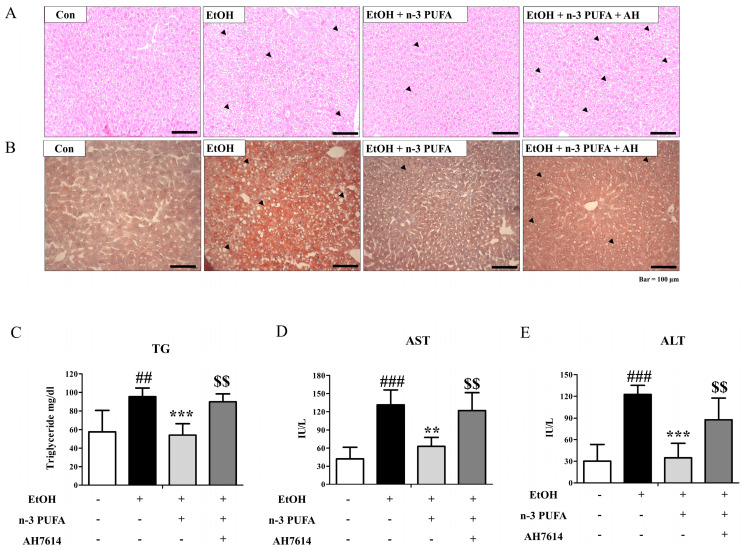
N-3 polyunsaturated fatty acids (n-3 PUFAs) protect against ethanol-induced hepatic steatosis. C57BL/6 female mice were fed a diet containing 6.3% ethanol with or without n-3 PUFAs (7.5 g/kg) and AH7614 (10 mg/kg) for two weeks. Livers were collected from mice fed a control diet (CD), EtOH diet, EtOH diet + n-3 PUFAs (6–7 animals per group), and EtOH diet + n-3 PUFAs + AH7614. (**A**) Hematoxylin and eosin (H&E)-stained liver sections showing liver morphology. Arrows indicate lesions of hepatic steatosis. (**B**) Oil red O staining. (**C**) Serum triglyceride levels. (**D**) Serum aspartate aminotransferase (AST) activities. (**E**) Serum alanine aminotransferase (ALT) activities. Data are presented as the mean ± standard error (SE) for 6 mice per experimental group. Statistical significance: ## *p* < 0.01 and ### *p* < 0.001 vs. CD-fed group; ** *p* < 0.01 vs. EtOH-fed group; *** *p* < 0.001 vs. EtOH-fed group; $$ *p* < 0.01 vs. EtOH- and n-3 PUFA-fed group contained in the second panel.

To assess the functions of n-3 PUFAs in ethanol-induced hepatic steatosis, eight-week-old C57BL/6 female mice were fed a liquid diet for one week and then fed a 6.3% ethanol-containing liquid diet for two weeks. N-3 PUFAs (7.5 g/kg) were simultaneously administered for two weeks (Supplemental Figure S1). Body weight did not change significantly after the two-week alcohol consumption. As shown in Figure 1A, a histological analysis of liver tissues showed the destruction of a well-organized structure of hepatocyte alignment by ethanol treatment and their recovery by n-3 PUFAs (Figure 1A). This effect of n-3 PUFA treatment was inhibited by AH7614 co-treatment. Because AH7614 is a competitive antagonist of FFA4, inhibition by AH7614 implies involvement of FFA4 in the action of n-3 PUFA. Oil red O staining showed that ethanol administration induced lipid accumulation (Figure 1B). The increase in Oil red O staining was attenuated by the administration of n-3 PUFAs (Figure 1B). Co-administration of AH7614 reversed the n-3 PUFA-induced inhibition of lipid accumulation (Figure 1B). Serum triglyceride levels were significantly increased in ethanol-treated mice (Figure 1C). This increase in triglyceride levels was significantly reduced by n-3 PUFAs, and AH7614 inhibited the effects of n-3 PUFAs (Figure 1C). Liver damage was monitored by measuring the aspartate aminotransferase (AST) and alanine aminotransferase (ALT) levels. As shown in Figure 1D,E, serum AST and ALT levels increased after ethanol administration. However, n-3 PUFA treatment reduced the serum levels of AST and ALT, indicating reduced liver damage (Figure 1D,E). AH7614 inhibited the protective effects of n-3 PUFAs, implying the involvement of FFA4 (Figure 1D,E). Therefore, n-3 PUFAs not only ameliorated ethanol-induced hepatic steatosis, as indicated by Oil red O staining, but also improved the ethanol-induced increase in serum triglyceride, AST, and ALT levels. However, AH7614 reversed the beneficial effects of n-3 PUFAs.

### 2.2. N-3 PUFAs Ameliorate Ethanol-Induced Changes in Lipogenic Gene Expression Levels in Mice

**Figure 2:**
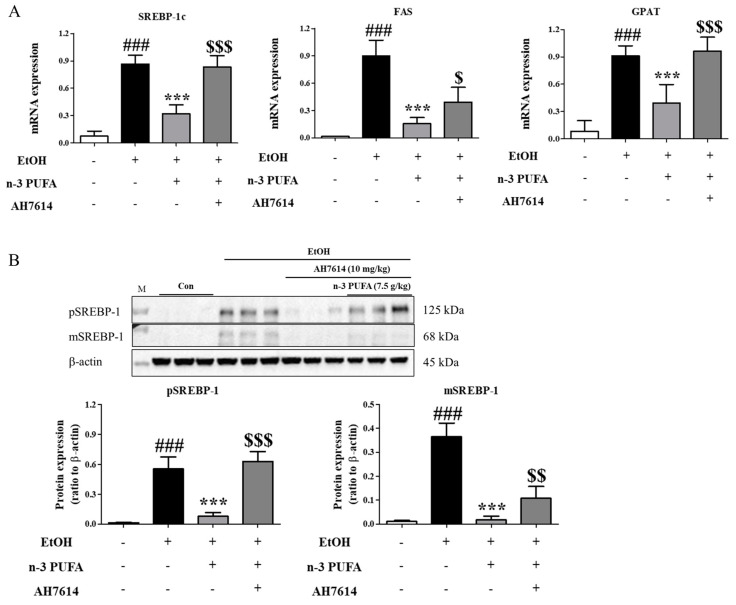
Modulation of lipogenic gene expression in the liver by n-3 PUFAs. (**A**) Reverse transcription-polymerase chain reaction (RT-PCR) analysis of the sterol regulatory element-binding protein-1c (*Srebp-1c*), fatty acid synthase (*Fas*), and glycerol-phosphate acyltransferase (*Gpat*) levels. (**B**) Western blotting of preformed and mature SREBP-1c expression levels. Data are presented as the mean ± SE. Data are presented as the mean ± standard error (SE) for 6 mice per experimental group. Statistical significance: ### *p* < 0.001 vs. CD-fed group; *** *p* < 0.001 vs. EtOH-fed group; $ *p* < 0.05, $$ *p* < 0.01, and $$$ *p* < 0.001 vs. EtOH-fed and n-3 PUFA-fed group.

Changes in the expression levels of lipogenesis-related genes in the liver were examined. SREBP-1c is a master regulatory transcription factor involved in de novo lipogenesis in the liver. Fatty acid synthase (FAS) and glycerol phosphate acyltransferase (GPAT) are involved in glycolysis and lipogenesis and promote triglyceride synthesis. As shown in Figure 2A, ethanol administration increased the mRNA expression levels of *Srebp-1c*, *Fas*, and *Gpat*, which correlated with hepatic steatosis observed in alcohol-treated mice. N-3 PUFAs reversed the increase in lipogenic gene expression, whereas AH7614 treatment reversed the effect of n-3 PUFAs on the expression levels of *Srebp-1c* and *Gpat* completely but *Fas* partially (Figure 2A). The effects of ethanol, n-3 PUFAs, and AH7614 on SREBP-1c expression levels were confirmed via Western blotting (Figure 2B). The full-length precursor SREBP-1 (preformed or pSREPB-1) is initially located in the endoplasmic reticulum. Upon activation signals, preformed SREBP-1 is transported to the Golgi apparatus and cleaved sequentially by proteases. The newly generated N-terminal fragment of SREBP (mature or mSREBP-1) translocates into the nucleus and stimulates lipogenic gene transcription. The levels of preformed and mature forms of SREBP-1c were high in ethanol-treated mice and decreased by n-3 PUFAs. Moreover, AH7614 reversed the effects of n-3 PUFAs (Figure 2B).

### 2.3. CpdA Ameliorates Ethanol-Induced Steatosis and Liver Injury in Mice

**Figure 3:**
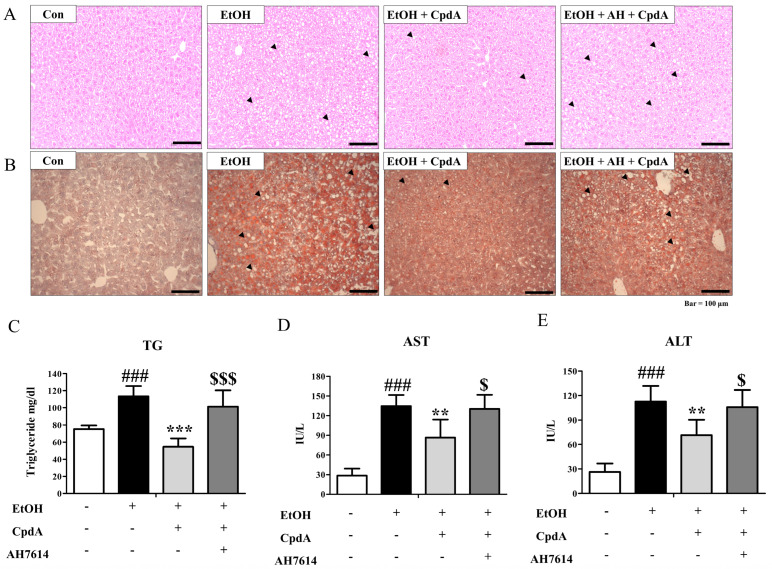
Compound A (CpdA) protects against ethanol-induced hepatic steatosis. C57BL/6 female mice were administered CpdA (30 mg/kg) and AH7614 (10 mg/kg) for two weeks. Livers were collected from mice fed CD, EtOH diet, EtOH diet + CpdA, and EtOH diet + CpdA + AH7614. (**A**) H&E-stained liver sections showing the liver morphology. Arrows indicate lesions of hepatic steatosis. (**B**) Oil red O staining. (**C**) Serum triglyceride levels. (**D**) Serum AST activities. (**E**) Serum ALT activities. Data are presented as the mean ± SE for 6 mice per experimental group. Statistical significance: ### *p* < 0.001 vs. CD-fed group; ** *p* < 0.01, *** *p* < 0.001 vs. EtOH-fed group; $ *p* < 0.05, $$$ *p* < 0.0001 vs. the EtOH-fed and CpdA-treated group.

To verify the involvement of FFA4 in the beneficial effects of n-3 PUFAs on ethanol-induced hepatic steatosis, CpdA (30 mg/kg) was simultaneously administered for two weeks (Supplemental Figure S1). As shown in Figure 3A, a histologic analysis of liver tissues showed that CpdA protected against ethanol-induced hepatocyte destruction (Figure 3A). Protection by CpdA treatment was reversed by AH7614 co-treatment. As shown in Figure 3B, CpdA administration attenuated the ethanol-induced increase in Oil red O staining in the liver. Co-administration with AH7614 reversed the CpdA-induced inhibition of lipid accumulation (Figure 3B). Serum triglyceride levels were significantly increased in ethanol-treated mice, but CpdA treatment significantly reduced these levels. Moreover, AH7614 reversed the effects of CpdA (Figure 3C). Ethanol-induced liver damage was monitored by measuring the levels of AST and ALT. The increased AST and ALT levels were protected by CpdA and reversed by AH7614 (Figure 3D,E). Therefore, CpdA ameliorated ethanol-induced hepatic steatosis (indicated by Oil red O staining) and improved the ethanol-induced increase in serum triglyceride, AST, and ALT levels, whereas AH7614 reversed these effects.

### 2.4. CpdA Ameliorates Ethanol-Induced Changes in Lipogenic Genes in Mice

**Figure 4:**
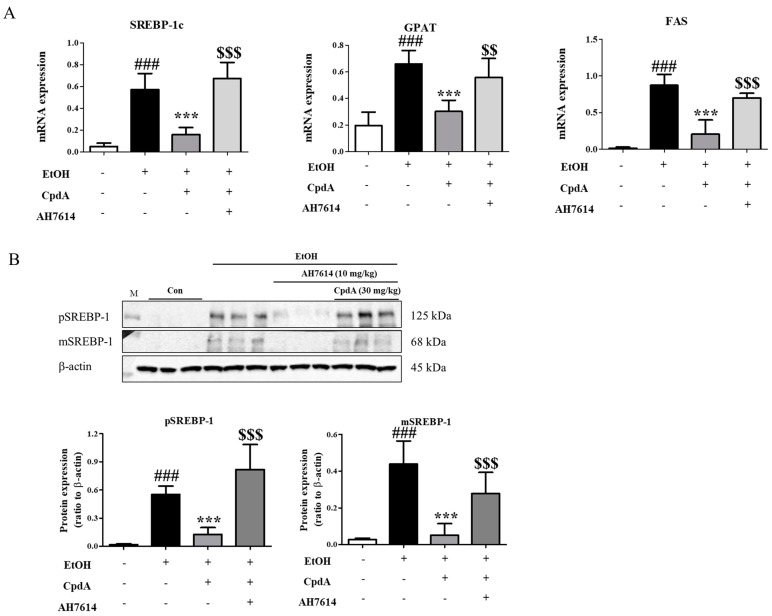
Modulation of lipogenic gene expression in the liver by CpdA. (**A**) RT-PCR analysis of *Srebp-1c*, *Fas*, and *Gpat* levels. (**B**) Western blotting of preformed and mature SREBP-1c expression levels. Data are presented as the mean ± SE for 6 mice per experimental group. Statistical significance: ### *p* < 0.001 vs. CD-fed group; *** *p* < 0.001 vs. EtOH-fed group; $$ *p* < 0.01 and $$$ *p* < 0.001 vs. EtOH-fed and CpdA-treated group.

Changes in the expression of lipogenesis-related genes were also examined in the liver. As shown in Figure 4A, CpdA administration inhibited the ethanol-induced increase in mRNA expression of *Srebp-1c*, *Fas*, and *Gpat*, whereas AH7614 reversed this effect (Figure 4A). The effects of ethanol, CpdA, and AH7614 on SREBP-1c expression were confirmed by Western blotting (Figure 4B). The increased expression of both the preformed and mature forms of SREBP-1c was suppressed by CpdA, whereas AH7614 reversed the effects of CpdA (Figure 4B).

### 2.5. Lack of Protective Effects of N-3 PUFAs or CpdA against Ethanol-Induced Steatosis and Liver Injury in FFA4 KO Mice

**Figure 5:**
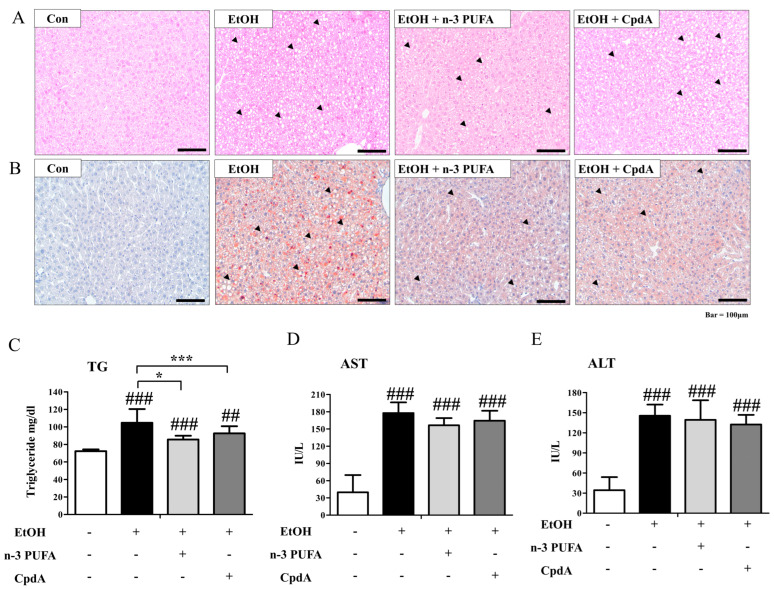
Effects of n-3 PUFA and CpdA on ethanol-induced hepatic steatosis in free fatty acid receptor 4 (FFA4) knockout (KO) mice. C57BL/6 female mice were fed a 6.3% ethanol diet containing n-3 PUFAs (7.5 g/kg) and CpdA (30 mg/kg) for two weeks. Livers were collected from mice fed CD, EtOH diet, EtOH diet + n-3 PUFAs, and EtOH diet + CpdA. (**A**) H&E-stained liver sections showing the liver morphology. Arrows indicate lesions of hepatic steatosis. (**B**) Oil red O staining. (**C**) Serum triglyceride levels. (**D**) Serum AST activities. (**E**) Serum ALT activities. Data are presented as the mean ± SE for 6 mice per experimental group. Statistical significance: ## *p* < 0.01, ### *p* < 0.001 vs. CD-fed group. * *p* < 0.1, *** *p* < 0.001 vs. EtOH-fed group.

To confirm the involvement of FFA4 in the functions of n-3 PUFAs against ethanol-induced hepatic steatosis, the same experiments were conducted using *Ffa4* KO mice. As shown in Figure 5A, a histological analysis of liver tissues revealed the destruction of hepatocytes by the ethanol treatment, but no significant improvement was observed with n-3 PUFAs or CpdA (Figure 5A). As shown in Figure 5B, ethanol-induced lipid accumulation was observed in *Ffa4* KO mice, and n-3 PUFA and CpdA treatments did not reverse the lipid accumulation. Serum triglyceride levels were significantly increased in the ethanol-treated *Ffa4* KO mice (Figure 5C), and this increase in triglyceride levels was significantly inhibited by n-3 PUFAs and CpdA (Figure 5C). However, the inhibition degrees by both n-3 PUFAs and CpdA were 37% and 57%, respectively, which are quite smaller than the complete inhibition in WT mice (Figure 1C and Figure 3C). Liver damage, monitored by serum AST and ALT levels, was increased by ethanol administration in *Ffa4* KO mice (Figure 5D,E). However, there was no significant suppression by n-3 PUFAs or CpdA in AST and ALT levels in *Ffa4* KO mice (Figure 5D,E). Therefore, there is significant suppression by n-3 PUFAs or CpdA in triglycerides, AST, or ALT levels in WT mice; however, these inhibitions were blunted in KO mice compared to the suppression degrees in WT mice. These results suggest that treatment with n-3 PUFA or CpdA ameliorates ethanol-induced hepatic steatosis or liver injury via FFA4. Comparing the levels of AST, ALT, and TG between WT and KO mice, there was a significant increase in the levels of AST in KO mice compared with WT mice with EtOH administration (Supplemental Figure S3). This may imply that deficiency of the Ffa4 gene resulted in loss of protective roles by endogenous Ffa4 activation.

### 2.6. Lack of Protective Effects of N-3 PUFAs or CpdA against Ethanol-Induced Changes in Srebp-1c Levels in Ffa4 KO Mice

**Figure 6:**
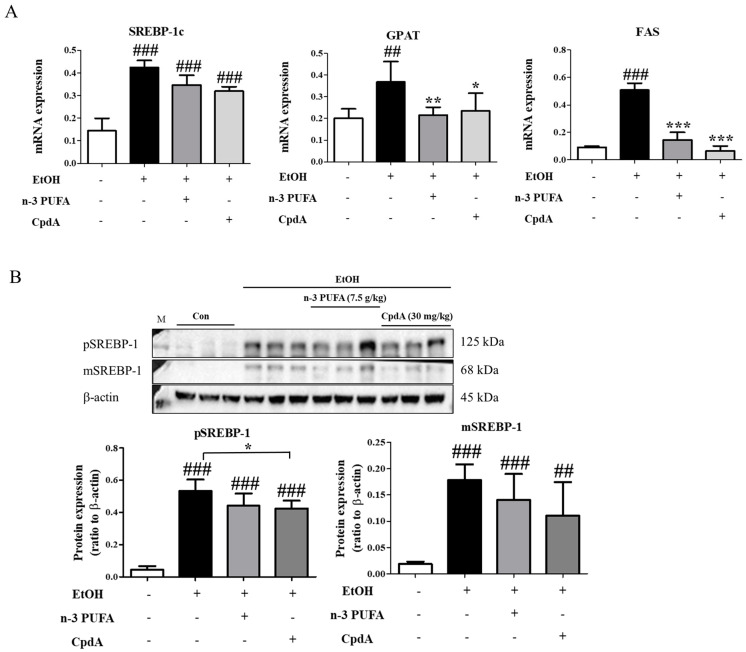
Effects of n-3 PUFAs and CpdA on lipogenic gene expression levels in the liver of FFA4 KO mice. (**A**) RT-PCR analysis of *Srebp-1c*, *Fas*, and *Gpat* levels. (**B**) Western blotting of preformed and mature SREBP-1c expression levels. Data are presented as the mean ± SE for 6 mice per experimental group. Statistical significance: ## *p* < 0.01, ### *p* < 0.001 vs. CD-fed group; * *p* < 0.05, ** *p* < 0.01, and *** *p* < 0.001 vs. EtOH-fed group.

Changes in the expression levels of genes related to lipogenesis were examined in the liver of *Ffa4* KO mice (Supplemental Figure S1). As shown in Figure 6A, ethanol administration increased the mRNA expression levels of *Srebp-1c*, *Fas*, and *Gpat* in *Ffa4* KO mice. Notably, n-3 PUFAs and CpdA did not reverse the increased expression of *Srebp-1c* but suppressed the expression of *Fas* and *Gpat* (Figure 6A). The effects of ethanol, n-3 PUFAs, and CpdA on SREBP-1c expression levels were confirmed via Western blotting (Figure 6B). Levels of preformed and mature SREBP-1c were increased in ethanol-treated *Ffa4* KO mice, and treatment with n-3 PUFAs or CpdA did not decrease SREBP-1c expression (Figure 6B).

Comparing the mRNA expression levels of *Srebp-1c*, *Fas*, and *Gpat* between WT and KO mice, there was a significant decrease in the expression levels of *Srebp-1c*, *Fas*, and *Gpat* mRNAs in KO mice compared with those in WT mice with EtOH administration but not without EtOH (Supplemental Figure S4A). However, at the protein levels, there was no significant change in preformed SREBP-1c, but there was a significant decrease in mature SREBP-1c after EtOH administration (Supplemental Figure S4B).

### 2.7. N-3 PUFAs and CpdA Inhibit Ethanol-Induced Activation of Kupffer Cells via FFA4

**Figure 7:**
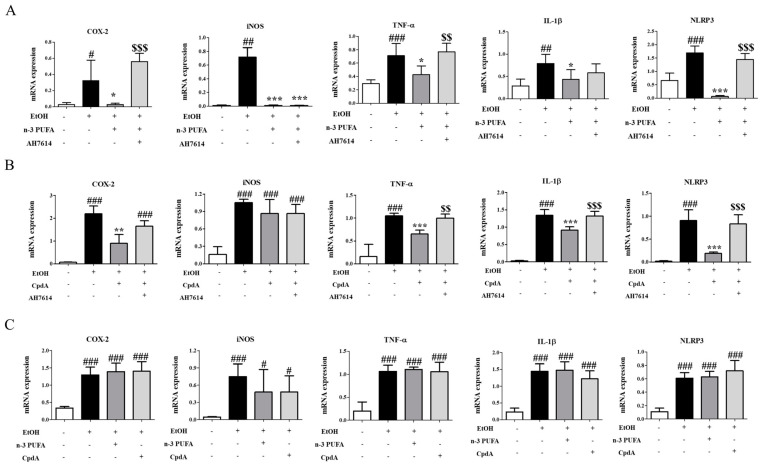
Change in pro-inflammatory gene expression levels in Kupffer cells by n-3 PUFAs, CpdA, and AH7614. RT-PCR analysis of pro-inflammatory genes. (**A**) Kupffer cells were collected from mice fed CD, EtOH diet, EtOH diet + n-3 PUFAs, and EtOH diet + n-3 PUFAs + AH7614. (**B**) Kupffer cells were collected from mice fed CD diet, EtOH diet, EtOH diet + CpdA, and EtOH diet + CpdA + AH7614. (**C**) Kupffer cells were collected from FFA4 KO mice fed CD diet, EtOH diet, EtOH diet + n-3 PUFAs, and EtOH diet + CpdA. Data are presented as the mean ± SE for 6 mice per experimental group. Statistical significance: # *p* < 0.05, ## *p* < 0.01, and ### *p* < 0.001 vs. CD-fed group; * *p* < 0.05, ** *p* < 0.01, and *** *p* < 0.001 vs. EtOH-fed group; $$ *p* < 0.01 and $$$ *p* < 0.001 vs. EtOH + n-3 PUFA or CpdA group.

As mentioned in the Introduction section, alcohol-induced steatosis is suggested to be mediated by the activation of Kupffer cells. Here, Ffa4 expression was assessed in isolated Kupffer cells using immunohistochemistry. Isolated Kupffer cells were confirmed by F4/80 staining (Supplemental Figure S2). Ffa4 expression was confirmed using a specific antibody, and all Kupffer cells were found to express Ffa4 (Supplemental Figure S2), consistent with a previous report [33]. Activation of Kupffer cells was assessed in CpdA-treated mice. High levels of cyclooxygenase (COX)-2, inducible nitric oxide synthase (*iNos*), *Tnf-α*, *Il-1β*, and NLR family pyrin domain-containing 3 (*Nlrp3*) were observed in the Kupffer cells of ethanol-treated mice (Figure 7A). Co-administration of n-3 PUFAs reduced the expression levels of these five genes, whereas AH-7614 reversed the expression levels of *Cox-2*, *Tnf-α*, and *Nlrp3* but not *iNos* and *Il-1β* (Figure 7A). Assessment of Kupffer cell activation was conducted in ethanol- and CpdA-treated mice in combination with AH7614 (Figure 7B). In the Kupffer cells of ethanol-treated mice, CpdA co-administration reduced the expression levels of *Cox-2*, *Tnf-α*, *Il-1β*, and *Nlrp3* but not *iNos*, whereas AH-7614 reversed the expression levels of *Cox-2*, *Tnf-α*, *Il-1*, and *Nlrp3* (Figure 7B). An assessment of Kupffer cell activation was further conducted in n-3 PUFA- and CpdA-treated *Ffa4* KO mice. In the Kupffer cells of ethanol-treated *Ffa4* KO mice, ethanol administration strongly induced the expression of *Cox-2*, *iNos*, *Tnf-α*, *Il-1β*, and *Nlrp3*. However, co-administration of n-3 PUFAs and CpdA did not ameliorate the increased expression levels of *Cox-2*, *iNos*, *Tnf-α*, *Il-1β*, and *Nlrp3* (Figure 7C), suggesting the involvement of FFA4.

## 3. Discussion

**Figure 8:**
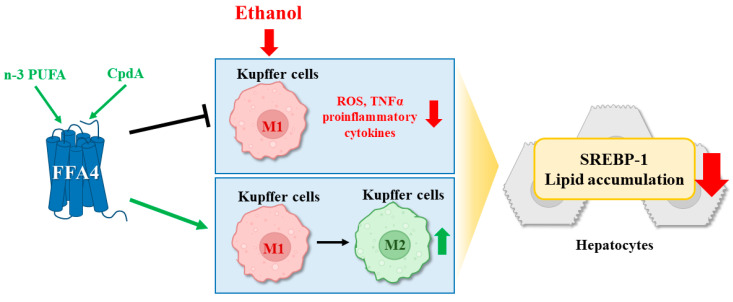
Illustration of the action mechanism of n-3 PUFAs and CpdA via Ffa4. EtOH administration disrupts the integrity of the intestinal epithelial layer and increases gut permeability, resulting in the exposure of endotoxins to Kupffer cells. Activation of Kupffer cells leads to the release of pro-inflammatory cytokines like TNF-α. Then, TNF-α induces SREBP-1, resulting in hepatic steatosis. FFA4 activation by n-3 PUFAs or CpdA induces suppression of pro-inflammatory M1 Kupffer cells and activation of anti-inflammatory M2 Kupffer cells, resulting in protection against steatosis.

In the present study, the protective effects of n-3 PUFAs and FFA4 against alcoholic liver steatosis were investigated. Activation of FFA4 by n-3 PUFAs inhibited hepatic steatosis, liver injury, expression of lipogenic SREBP-1c in alcohol-treated mice, and expression of *Tnf-α*, *Cox-2*, and *Nlrp3* in Kupffer cells (Figure 8). Previous studies support the protective effects of n-3 PUFAs against non-alcoholic fatty liver [8,9,36,37,38,39]. Fish oil decreases mature SREBP-1 levels by downregulating *Srebp-1c* mRNA expression in the mouse liver. These effects are mainly due to the combined effects of inhibition of lipogenesis via inactivation of SREBP-1c in the liver [39,40]. N-3 PUFAs move away from lipid synthesis (triglyceride production) and toward fatty acid oxidation in non-alcoholic fatty liver disease [9,41]. Therefore, dietary fish oil may be useful for preventing alcohol-induced fatty liver disease. However, the pathogenesis of alcoholic steatosis is somewhat different from that of non-alcoholic hepatic steatosis.

The mechanisms underlying chronic ethanol-induced steatosis have been extensively studied. Increases in the mature (active) form of SREBP-1 and the expression of its target lipogenic genes have been reported in C57BL/6J mice fed ethanol as a liquid diet for 4 weeks [42]. The predominant role of SREBP-1c in hepatic steatosis has also been reported in *Srebp-1c*-null mice fed ethanol via intragastric infusion for 4 weeks [43]. The protective effects of n-3 PUFAs are mediated by decreased SREBP-1c activity [44] or reduced oxidative/nitrosative stress in alcohol-induced steatosis [14]. In an acute model, ethanol-induced hepatic steatosis was induced in nine-week-old male mice via oral gavage of ethanol (4.7 g/kg BW) with or without DHA (250 mg/kg BW) every 12 h for three administrations [15]. DHA suppressed acute ethanol-induced hepatic steatosis and downregulated the levels of stearoyl-CoA desaturase 1 (SCD) and inflammatory cytokines, such as TNF-α and IL-6, but did not affect reactive oxygen species production [15]. In a chronic alcohol-induced steatosis model, male Long Evans rats were fed an ethanol or control liquid diet with or without low levels of arachidonic acid (AA) and DHA (0.56 gm each/L) liquid diet for 9 weeks. Chronic alcohol administration increased the degree of fatty liver, but the addition of DHA/AA prevented alcohol-induced fatty liver and mitochondrial dysfunction [14]. Acute ethanol-induced hepatic steatosis was alleviated, and the hepatic expression of SREBP-1c and plasma levels of TNF-α, IL-6, and MCP-1 were reduced in fat-1 transgenic mice [16].

In previous studies, the protective roles of n-3 PUFAs in ethanol-induced hepatic steatosis have been inconsistent. Fish oil promotes the pathogenesis of ethanol-induced liver injury and retards ethanol metabolism [45,46,47]. Markedly increased CYP 2E1 induction and lipid peroxidation have been proposed as mechanisms of ethanol-induced liver injury [46,47]. However, these negative effects were possibly due to poor-quality fish oil, as fish oil contains highly unstable long-chain n-3 PUFAs, and easily oxidized products can cause negative effects [15,48]. In this study, clinically used highly purified n-3 PUFAs reproduced the protective effects of n-3 PUFA against alcoholic hepatic steatosis. Our finding suggests that the suppression of Kupffer cell activation by FFA4 contributes to the protective functions of n-3 PUFAs (Figure 8). In fact, strong expression of Ffa4 in F4/80-positive Kupffer cells is confirmed by immunohistochemistry in the liver tissue section of WT mice after EtOH administration (Supplemental Figure S5). Without EtOH treatment, Ffa4 is very weakly expressed in hepatocytes widely, but its expression also increased in hepatocytes after EtOH administration (Supplemental Figures S5 and S6), supporting the protective functions of Ffa4 in hepatocytes against non-alcoholic hepatic steatosis [49].

In the present two-week alcoholic liquid diet model, ethanol induced lipid accumulation and SREBP-1c expression. The hepatic steatosis response in ethanol-treated mice was completely reversed by the co-administration of n-3 PUFAs and CpdA, consistent with previous reports [15,16]. In addition, n-3 PUFA and CpdA administration reduced the ethanol-induced increase in serum triglyceride levels. However, there are several limitations to this study. First, we have not investigated whether n-3 PUFAs and CpdA reduce oxidative/nitrosative stress and mitochondrial dysfunction in alcohol-induced steatosis in an FFA4-dependent manner. Second, we were not able to observe positive effects of n-3 PUFAs and CpdA on fatty acid oxidation, such as PPAR-α. Third, the present study should be further investigated in a chronic alcohol-induced steatosis model such as 9 weeks.

Changes in the expression of lipogenic SREBP-1c in hepatocytes are partly mediated by the indirect activation of Kupffer cells. The importance of bacterial endotoxins in the intestine, Kupffer cell activation, and TNF-α secretion has been demonstrated by gut sterilization, Kupffer cell depletion, and *TNF-R1* deficiency [21,22,23,24,25,26,27,28]. Ethanol administration increased the expression levels of inflammatory marker genes, such as *Tnf-α*, *Cox-2*, *iNos*, *Il-1β*, and *Nlrp-3*, in Kupffer cells. These inflammatory responses in the Kupffer cells of ethanol-treated mice were completely suppressed by the co-administration of n-3 PUFAs and CpdA, supporting our hypothesis (Figure 8).

FFA4 is highly expressed in Kupffer cells of the liver [33]. The functional roles of FFA4 in Kupffer cells have been investigated in an ischemia-reperfusion injury model [33]. An FFA4 agonist and Omegaven^®^, a clinically used intravenous formulation of n-3 PUFA, protected against ischemia-reperfusion injury, which was abolished by clodronate-depletion of Kupffer cells or pretreatment with siRNA against FFA4 [33]. Here, administration of AH7614 ameliorated n-3 PUFA- or CpdA-induced protection against alcoholic hepatic steatosis and inactivation of Kupffer cells. Similarly, the protective effects of n-3 PUFAs and CpdA against alcoholic hepatic steatosis and Kupffer cell activation were not observed in *Ffa4* KO mice. Considering the key roles of Kupffer cell activation and inflammation in alcoholic liver steatosis, n-3 PUFA- and CpdA-mediated Ffa4 activation in Kupffer cells may lead to the suppression of pro-inflammatory genes, resulting in an alleviation of alcoholic liver steatosis.

To the best of our knowledge, the present study is the first to demonstrate the functional roles of FFA4 in alcoholic steatosis. Blocking FFA4 by AH7614 or *Ffa4* deficiency ameliorated n-3 PUFA-induced protection against alcoholic hepatic steatosis and pro-inflammatory gene expression in Kupffer cells. Indeed, n-3 PUFAs act on FFA4 in Kupffer cells, protecting against alcoholic steatosis in the liver.

In conclusion, this study demonstrated the protective effects of n-3 PUFAs against hepatic steatosis via FFA4. Our finding suggests the activation of FFA4 as a key mechanism underlying the beneficial protective effects of n-3 PUFAs against alcoholic fatty liver disease.

## 4. Materials and Methods

### 4.1. Materials

Omacor^®^, clinically prescribed high-purity n-3 PUFAs, was provided by Kunil Pharmaceuticals (Seoul, Republic of Korea). CpdA (Cat no. 16624) was purchased from Cayman (Ann Arbor, MI, USA). AH7614 was obtained from Tocris Bioscience (Cat. no. 5256/10, Ellisville, MO, USA).

### 4.2. Animals, Diets, and Treatment

*Ffa4* knockout mice (TF0224) were purchased from Lexicon Pharmaceuticals (Woodlands, TX, USA) and backcrossed to C57BL/6 mice for 8 generations [49,50,51]. All animals were housed in a laboratory animal facility at Pusan National University and provided with food and water ad libitum. All animal procedures were conducted in compliance with the guidelines for animal care and use of the Pusan National University. The experimental protocol was evaluated and approved by the Institutional Animal Care and Use Committee of Pusan National University with respect to the ethics of the procedures and care provided (Approval Number PNU-2016-1173). Genotyping was conducted by PCR using primer pairs as follows: WT forward 5′-GAG CGC ATG GTG CAT CG-3′, WT reverse 5′-CAC GGC TTT GGT CAG ATC C-3′, KO forward 5′-GCA GCG CAT CGC CTT CTA TC-3′, and KO reverse 5′-TTG GCA CTG TGG GTA AAC TGA CGA-3′.

Eight-week-old C57BL/6 female mice were divided into 12 groups (n = 6) as follows: (1) control diet (CD)-fed FFA4 WT, ethanol-fed FFA4 WT, ethanol-fed n-3 PUFAs-treated FFA4 WT, n-3 PUFAs/AH7614-treated FFA4 WT (2) CD-fed FFA4 WT, ethanol-fed FFA4 WT, ethanol-fed compound A-treated FFA4 WT, ethanol-fed compound A/AH7614-treated FFA4 WT (3) CD-fed FFA4 KO, ethanol-fed FFA4 KO, ethanol-fed n-3 PUFAs-treated FFA4 KO, and ethanol-fed compound A-treated FFA4 KO group. Mice were adapted to liquid diets for one week and exposed to a 6.3% ethanol-containing liquid diet for two weeks [52]. N-3 PUFAs (7.5 g/kg) were simultaneously administrated for two weeks in Lieber-De Carli (Supplemental Figure S1) [52]. The composition of the high-fat liquid diet was 56% carbohydrate, 28% fat, and 16% protein [52]. The final concentration of ethanol in this liquid diet was 6.3% (*v*/*v*), and ethanol accounts for 28% of the total caloric intake (Table 1) [19]. The control diet (CD) was obtained by replacing the ethanol with an equivalent quantity of maltodextrin [19]. During the two weeks of feeding with ethanol, CpdA (30 mg/kg) was injected daily i.p., and AH7614 was administered by i.p. injections every 2 days.

### 4.3. Measurement for ALT, AST, and TG

Blood samples were collected in tubes and immediately centrifuged at 900× *g* for 10 min at 4 °C. ALT, AST, and TG levels were measured using measurement kits (Cat No. AM102-k, AM103-k, and AM157S-k; Asan Pharmaceutical, Seoul, Republic of Korea).

### 4.4. Isolation of Primary Kupffer Cells

Mouse livers were perfused with HBSS (Cat No. 14065-056; Gibco, Grand Island, NY, USA), containing 0.05% collagenase (Cat No. 17101-015; Gibco, Grand Island, NY, USA). By centrifugation of 50× *g* for 1 min, hepatocytes were pelleted, and the supernatant containing non-parenchymal cells were resuspended in RPMI (Cat No. 11875093, Gibco, Grand Island, NY, USA) with fetal bovine serum (FBS, Cat No. 26140079, Gibco, Grand Island, NY, USA) and separated by centrifugation (200× *g* for 20 min) on a 25–50% Percoll gradient (Cat No. 17-0891-01, GE Healthcare Life Sciences, Marlborough, MA, USA) [19]. The Kupffer cells were seeded in RPMI containing 10% FBS.

### 4.5. Reverse Transcription-Polymerase Chain Reaction (RT-PCR)

We evaluated the expression levels of inflammatory markers in Kupffer cells and lipid accumulation in the mouse livers using RT-PCR. First-strand cDNA was synthesized from 1 μg of total RNA isolated using Trizol reagent (Invitrogen, Waltham, MA, USA) using Oligo (dT) 15 primer and M-MLV reverse transcriptase (Promega, Madison, WI, USA). Synthesized cDNA products (1 μL), primers for each gene, and Promega Go-Taq DNA polymerase (Cat No. M3001, Promega, Madison, WI, USA) were used for PCR. Sequences of primers were summarized in Table 2. PCR was performed using 25 or 27 cycles at 95 °C for 1 min (denaturation), 60 °C for 1 min (annealing), and 72 °C for 2 min (elongation) in an Eppendorf Mastcyclergradient PCR machine (Hamburg, Germany). Aliquots of the PCR products (7 μL) were electrophoresed in 1.2% agarose gels, and the gels were stained with Nucleic acid gel stain (Real Biotech, Taipei, Taiwan) [53].

### 4.6. Western Blot Analysis

The total protein isolation from the cells was scraped into lysis buffer. The concentrations of the proteins were determined using a BCA protein assay (Cat No. 23225, Thermo Scientific, Rockford, IL, USA), and equal amounts of proteins were resolved by 10% SDS-polyacrylamide gel electrophoresis and electrophoretically transferred to nitrocellulose membranes. The membranes were blocked in Tris-buffered saline containing 0.1% Tween 20 (TBS-T) and 5% skim milk and then incubated with a specific rabbit antibody recognizing SREBP-1c (Cat No. sc-366, Santa Cruz Biotechnology, Dallas, TX, USA). Anti-rabbit horseradish peroxidase-linked IgG (Cat No. 7074, Cell Signaling Technology, Danvers, MA, USA) was used as the secondary antibody. Signals were developed using an enhanced chemiluminescence system (Cat No. K-12045-D50, Advantest, San Jose, CA, USA) [49].

### 4.7. Histology

Specimens from the liver were fixed in 10% formalin (Cat No. FR1043-100-01, Biosesang, Yongin, Gyeong-gi-do, Republic of Korea) for 48 h and dehydrated in a 30% sucrose solution for 24 h at 4 °C. Liver tissue samples were embedded in an O.C.T. compound and frozen. Liver cryosections (5 μm) were stained with hematoxylin (Cat No. S3309, DAKO, Santa Clara, CA, USA) and eosin (Cat No. CS701, DAKO, Santa Clara, CA, USA) (H&E) and Oil red O (Cat No. O1391, Sigma-Aldrich, Saint Louis, MO, USA) [49,50,51].

### 4.8. Statistics

The results are expressed as the mean ± SE for the number of indicated determinations. Differences among treatment groups were investigated using analysis of variance (ANOVA) with Tukey’s multiple comparison post-hoc test, and statistical significance was accepted for *p* values < 0.05.

## Figures and Tables

**Table 1:** Composition of the liquid diet.

Ingredients	g/L (Con)	g/L (EtOH)
Casein hydrolysate	53	53
Dextri-maltose	177	85
AIN-76 mineral mix	9.3	9.3
AIN-76A vitamin mix	2.7	2.7
DL-Methionine	0.8	0.8
Choline bitartrate	0.5	0.5
Xanthan gum	3	3
Alphacel non-nutritive bulk	13	13
Corn oil	8.5	8.5
Safflower oil	2.7	2.7
Olive oil	28.4	28.4

**Table 2:** Primer sequences.

Gene	Sequence
*mCox-2*	F: 5′-CCGTGGGGAATGTATGAGCA-3′ R: 5′-CCAGGTCCTCGCTTATGATCTG-3′
*mIl-1β*	F: 5′-GGAGAAGCTGTGGCAGCTA-3′ R: 5′-GCTGATGTACCAGTTGGGGA-3′
*mTNF-α*	F: 5′-TGAGCACAGAAAGCATGACC-3′ R: 5′-AGGGTCTGGGCCATAGAACT-3′
*mNlrp3*	F: 5′-CCTTGGACCAGGTTCAGTGT-3′ R: 5′-AGGAGATGTCGAAGCAGCAT-3′
*miNos*	F: 5′-ACCTACCACACCCGAGATGGCCAG-3′ R: 5′-AGGATGTCCTGAACATAGACCTTGGG-3′
*mSrebp-1c*	F: 5′-GCGCTACCGGTCTTCTATCA-3′ R: 5′-TGCTGCCAAAAGACAAGGG-3′
*mFas*	F: 5′-TGGGTTCTAGCCAGCAGAGT-3′ R: 5′-ACCACCAGAGACCGTTATGC-3′
*mGpat*	F: 5′-AGCAAGTCCTGCGCTATCAT-3′ R: 5′-CTCGTGTGGGTGATTGTGAC-3′
*mGapdh*	F: 5′-GCGCTACCGGTCTTCTATCA-3′ R: 5′-TGCTGCCAAAAGACAAGGG-3′

## Data Availability

Data are available on requests.

## Supplementary Materials

The supporting information can be downloaded at https://www.mdpi.com/article/10.3390/ijms25105476/s1.

## Abbreviations

AA, arachidonic acid; ALT, alanine aminotransferase; AST, aspartate aminotransferase; BW, body weight; CD, control diet; CpdA, compound A; DHA, docosahexaenoic acid; FAS, fatty acid synthase; FFA4, free fatty acid receptor 4; GPAT, glycerol phosphate acyltransferase; iNOS, inducible nitric oxide synthase; LPS, lipopolysaccharides; NLRP3, NLR family pyrin domain-containing 3; mSREBP, mature SREBP-1; TNF-α, tumor necrosis factor-α; pSREBP-1, preformed SREPB-1; PPAR-α, peroxisome proliferator-activated receptor-α; PUFA, polyunsaturated fatty acid; RT-PCR, reverse transcription-polymerase chain reaction; SCD, stearoyl-CoA desaturase; SREBP-1c, sterol regulatory element-binding protein-1c.
