# Supplementary material for: N-3 Polyunsaturated Fatty Acids Protect against Alcoholic Liver Steatosis by Activating FFA4 in Kupffer Cells
Source: Int J Mol Sci. 2024 May 17;25(10):5476. doi: 10.3390/ijms25105476 (PMC11122576; doi:10.3390/ijms25105476)
Supplement: Supplementary file 1 [file ijms-25-05476-s001.zip › ijms-2978199-supplementary.pdf]

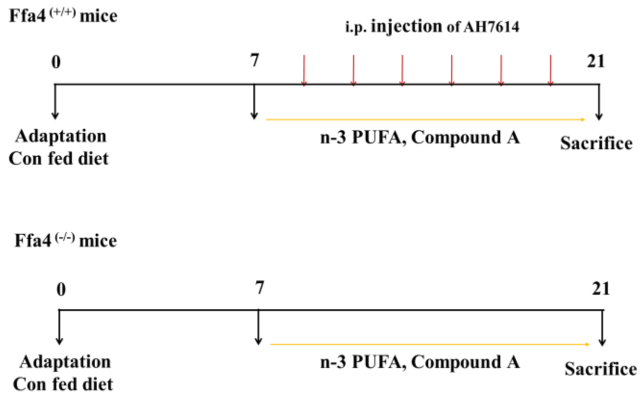

**Agonist**	n-3 PUFA 7.5g/kg
	Compound A 30 mg/kg
**Antagonist**	AH 7614 10 mg/kg

Figure S1. Experimental scheme. Eight-week old C57BL/6 female mice were adapted to liquid diets for one week and exposed to 6.3% ethanol-containing liquid diet for two weeks [52]. N-3 PUFAs (7.5 g/kg) were simultaneously administrated for two weeks in Lieber-De Carli (Supplemental Figure-1)[52]. The composition of the high-fat liquid diet was 56% carbohydrate, 28% fat, and 16% protein [52]. The final concentration of ethanol in this liquid diet was 6.3% (vol/vol), and ethanol accounts for 28% of total caloric intake (Table 1) [19]. The control diet (CD) was obtained by replacing the ethanol by an equivalent quantity of maltodextrin [19]. CpdA was administrated by i.p. injection every day. AH7614 was administrated by i.p. injection every 2 days

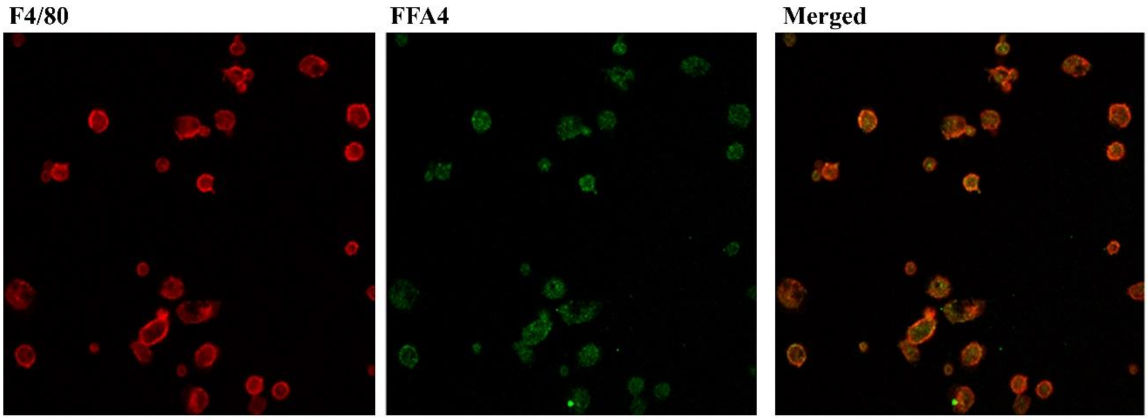

Figure S2. Fluorescence confocal microscopy of Kupffer cells. Isolated Kupffer cells were labeled with antibodies against FFA4 (NBPI-00858, 1:500, Novus Biologicals, LLC, CO, USA), and F4/80 (ab6640, 1:100, abcam, Cambrigde, MA, USA). The secondary antibody used was Alexa Flour 488-conjugated anti-rabbit IgG (1:8000, Jackson ImmunoResearch, West Grove, PA, USA) and Cy™-3-conjugated anti-rat 1gG (1:8000, Jackson ImmunoResearch, West Grove, PA, USA). A confocal scanning module (LSMS10, Carl Zeiss, Germany) mounted on a fluorescence microscope (AXIOVERT 100 M, Carl Zeiss, Germany) using a C-Apochromat 40x/1.2 W (Carl Zeiss, Germany) was used (Kim JM et al., 2015)

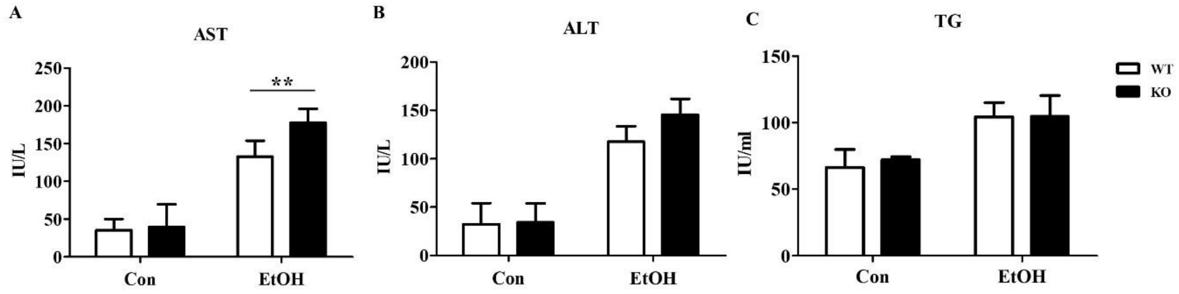

Figure S3. Comparison of AST, ALT, and TG levels between WT and KO mice. The serum levels of AST, ALT, and TG from WT and KO mice without treatment or with treatment of EtOH were compared. (A) AST. (B) ALT. (C) TG levels. Data are presented as the mean ± standard error (SE) for 6 mice per experimental group. Statistical significance: ** $p<0.01$ vs. WT

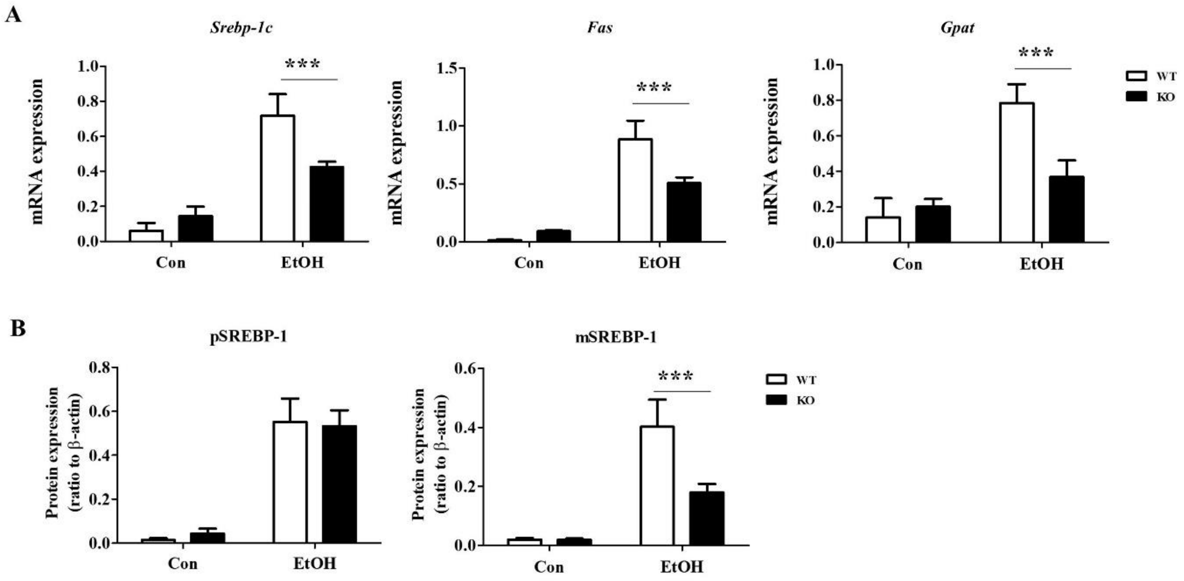

Figure S4. Comparison of mRNA expression levels of *Srebp-1c*, *Fas*, and *Gpat* and protein levels of pSrebp-1c and mSrebp-1c between WT and KO mice. (A) The expression levels of *Sehp-1c*, *Fas*, and *Gpat* mRNAs between WT and KO mice without treatment or with treatment of EtOH were compared. (B) The expression levels of preform and mature forms of Srebp-1c between WT and KO mice without treatment or with treatment of EtOH were compared. Date are presented as the mean ± standard error (SE) for 6 mice per experimental group. Statistical significance: *** $p<0.001$ vs. WT mice

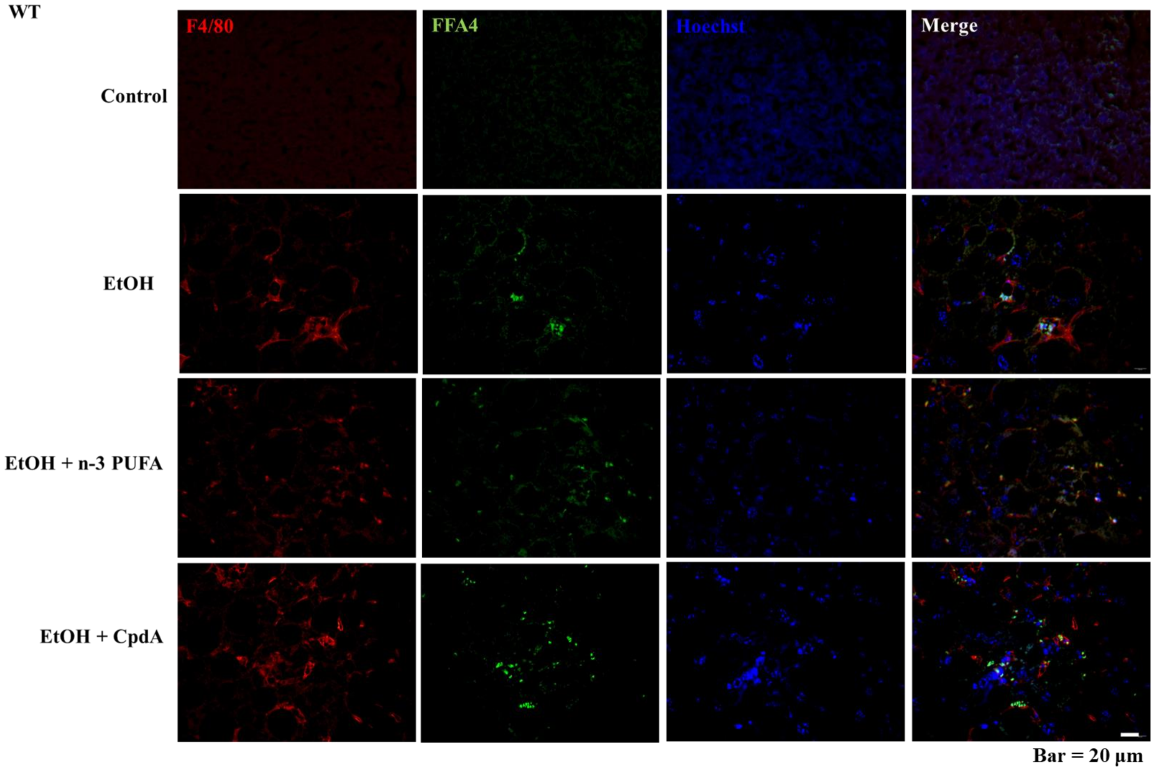

Figure S5. Immunohistochemistry of F4/80 and Ffa4 in liver tissue section of WT mice. (Red color) Macrophages were stained with F4/80 antibody (ab6640, 1:100, abcam, Cambridge, MA, USA). (Green color) Ffa4 was identified with a specific antibody (NBP1-00858, 1:500, Novus Biologicals, LLC, CO, USA). (Blue color) Hoechst staining shows nuclei.

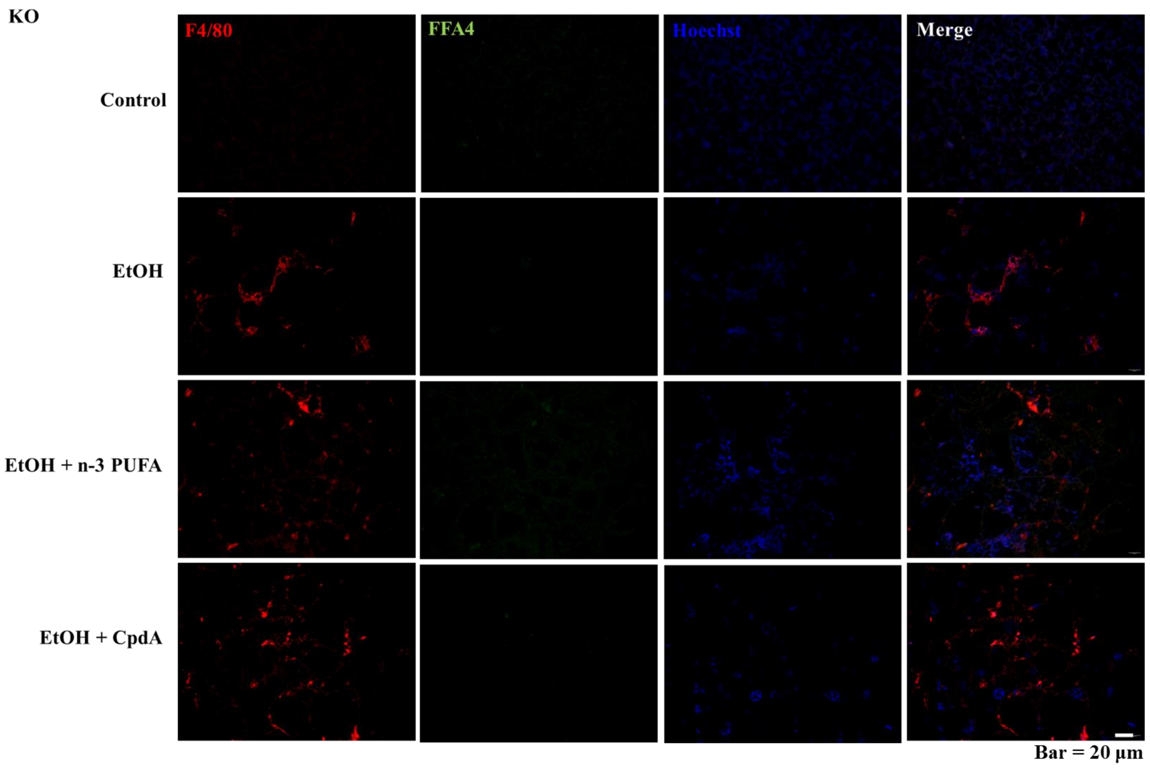

Figure S6. Immunohistochemistry of F4/80 and Ffa4 in liver tissue section of KO mice. (Red color) Macrophages were stained with F4/80 antibody (ab6640, 1:100, abcam, Cambridge, MA, USA). (Green color) Ffa4 was identified with a specific antibody (NBP1-00858, 1:500, Novus Biologicals, LLC, CO, USA). (Blue color) Hoechst staining shows nuclei.
